# Ultrasonic assessment of exercise-induced change in skeletal muscle glycogen content

**DOI:** 10.1186/s13102-015-0003-z

**Published:** 2015-04-18

**Authors:** David C Nieman, R Andrew Shanely, Kevin A Zwetsloot, Mary Pat Meaney, Gerald E Farris

**Affiliations:** 1 Appalachian State University, Human Performance Lab, North Carolina Research Campus, 600 Laureate Way, Kannapolis, NC 28081 USA; 2 Department of Health and Exercise Science, Appalachian State University, Boone, NC USA; 3 Department of Emergency Medicine, Carolinas Medical Center NorthEast, Concord, NC USA

**Keywords:** Cycling, Muscle biopsy, Vastus lateralis, Skeletal muscle, Sonography

## Abstract

**Background:**

Ultrasound imaging is a valuable tool in exercise and sport science research, and has been used to visualize and track real-time movement of muscles and tendons, estimate hydration status in body tissues, and most recently, quantify skeletal muscle glycogen content. In this validation study, direct glycogen quantification from pre-and post-exercise muscle biopsy samples was compared with glycogen content estimates made through a portable, diagnostic high-frequency ultrasound and cloud-based software system (MuscleSound®, Denver, CO).

**Methods:**

Well-trained cyclists (N = 20, age 38.4 ± 6.0 y, 351 ± 57.6 watts_max_) participated in a 75-km cycling time trial on their own bicycles using CompuTrainer Pro Model 8001 trainers (RacerMate, Seattle, WA). Muscle biopsy samples and ultrasound measurements were acquired pre- and post-exercise. Specific locations on the vastus lateralis were marked, and a trained technician used a 12 MHz linear transducer and a standard diagnostic high resolution GE LOGIQ-e ultrasound machine (GE Healthcare, Milwaukee, WI) to make three ultrasound measurements. Ultrasound images were pre-processed to isolate the muscle area under analysis, with the mean pixel intensity averaged from the three scans and scaled (0 to 100 scale) to create the glycogen score. Pre- and post-exercise muscle biopsy samples were acquired at the vastus lateralis location (2 cm apart) using the suction-modified percutaneous needle biopsy procedure, and analyzed for glycogen content.

**Results:**

The 20 cyclists completed the 75-km cycling time trial in 168 ± 26.0 minutes at a power output of 193 ± 57.8 watts (54.2 ± 9.6% watts_max_). Muscle glycogen decreased 77.2 ± 17.4%, with an absolute change of 71.4 ± 23.1 mmol glycogen per kilogram of muscle. The MuscleSound® change score at the vastus lateralis site correlated highly with change in measured muscle glycogen content (R = 0.92, P < 0.001).

**Conclusions:**

MuscleSound® change scores acquired from an average of three ultrasound scans at the vastus lateralis site correlated significantly with change in vastus lateralis muscle glycogen content. These data support the use of the MuscleSound® system for accurately and non-invasively estimating exercise-induced decreases in vastus lateralis skeletal muscle glycogen content.

## Background

Muscle glycogen content is important for high-intensity exercise, and low levels are related to fatigue [1]. Muscle glycogen content is typically analyzed in research settings using muscle samples obtained with percutaneous biopsy needles, imposing significant participant burden in terms of discomfort and time, especially when repeated measurements are made [2,3]. Magnetic resonance spectroscopy (MRS) is used to non-invasively measure tissue glycogen using: 1) ^13^C natural abundance levels, or ^13^C atoms incorporated into glycogen by ^13^C substrate received through ingestion or intravenous administration; and 2) the water signal with chemical exchange saturation transfer imaging (glycoCEST) [4-6]. These MRS techniques involve significant investments in terms of equipment expenditure and technician training, and are not available in portable form for use in athletic settings.

Ultrasound or sonography is widely used in medicine, and has several advantages compared to other prominent methods of imaging including portability, low cost, the absence of harmful ionizing radiation, the provision of images in real-time, no discomfort or long-term side effects to the participant, and widely available equipment. In exercise and sport science research, ultrasound imaging is used for a wide variety of applications including evaluation of the cardiovascular status of athletes, musculoskeletal pathology diagnosis and therapeutic interventions, and to visualize and track real-time movement of muscles and tendons [7,8]. The ultrasonographic image of muscles is distinct and can easily be discriminated from surrounding tissues such as bone, nerves, blood vessels, and subcutaneous fat [9]. Ultrasound velocity can be used to assess hydration status in body tissues including muscle that contains 70-80% water [10,11], and detect structural muscle changes caused by neuromuscular disease [12].

MuscleSound® utilizes portable, diagnostic high-frequency ultrasound technology and cloud-based software to non-invasively measure change in muscle glycogen content. This methodology is based upon measurement of the water content associated with glycogen in muscle. When muscle glycogen content is high, the ultrasound image is hypoechoic (dark), and with glycogen depletion and water loss, the image is hyperchoic (brighter). The MuscleSound® software quantifies change in muscle glycogen content using image processing and analysis through segmentation of the region of interest and measurement of the mean signal intensities. One previous study using muscle biopsy samples taken from the rectus femoris in 22 cyclists before and after 90 minutes of steady-state cycling showed a correlation of 0.81 between the modest change in muscle glycogen content and the glycogen change score calculated with MuscleSound® technology [13]. Muscle biopsy samples are typically taken from the vastus lateralis, and the present study extended these results by comparing estimation of change in muscle glycogen content from the MuscleSound® device with direct glycogen content quantification from pre- and post-exercise muscle biopsies taken from the vastus lateralis muscles of cyclists participating in a 75-km cycling time trial.

## Methods

### Subjects and baseline testing

Subjects included 20 male cyclists (ages 18 to 55 y) who regularly competed in road races and had experience with long distance cycling time trials. Subjects voluntarily provided informed consent and all study procedures were approved by the Institutional Review Board at Appalachian State University. One week prior to the 75-km time trial, each athlete completed orientation/baseline testing in the North Carolina Research Campus Human Performance Laboratory operated by Appalachian State University in Kannapolis, NC. Demographic and training histories were acquired with questionnaires. During baseline testing, maximal power, oxygen consumption, ventilation, and heart rate were measured during a graded exercise test (25 Watts increase every two minutes, starting at 150 Watts) with the Cosmed Quark CPET metabolic cart (Rome, Italy) and the Lode cycle ergometer (Lode Excaliber Sport, Lode B.V., Groningen, Netherlands). Body composition was measured with the Bod Pod body composition analyzer (Life Measurement, Concord, CA).

### 75-km cycling time trial

One week following baseline testing, subjects participated in a 75-km cycling time trial on their own bicycles on CompuTrainer Pro Model 8001 trainers (RacerMate, Seattle, WA). A mountainous 75-km course with moderate difficulty was chosen and programmed into the software system. Heart rate and rating of perceived exertion (RPE) were recorded at 15 minutes, and every 60 minutes thereafter, and workload in watts was continuously monitored using the CompuTrainer MultiRider software system (version 3.0). Oxygen consumption and ventilation were measured using the Cosmed Quark CPET metabolic cart (Rome, Italy) after 16 km and 55 km cycling. Subjects were allowed to ingest water ad libitum during the 75-km cycling time trial.

### Blood sample analysis

Blood samples were collected pre- and post-exercise and analyzed for plasma glucose, plasma lactate, serum cortisol, and serum myoglobin. Plasma glucose and lactate were analyzed using the YSI 2300 STAT Plus Glucose and Lactate analyzer (Yellow Springs, OH). Serum myoglobin was measured using an LX-20 clinical analyzer (Beckman Coulter Electronics, Brea, CA), and cortisol with an electrochemiluminescence immunoassay (ECLIA) through a commercial lab (LabCorp, Burlington, NC).

### Skeletal muscle ultrasound procedures

**Figure 1:**
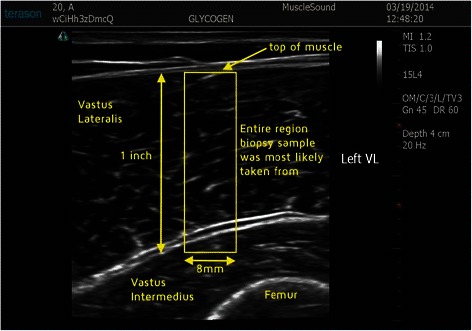
Ultrasonic scan from a subject with the rectangle area representing where images were segmented to isolate the muscle area under analysis using a center crop within the muscle section 25 mm from the top of the muscle sheath.

**Figure 2:**
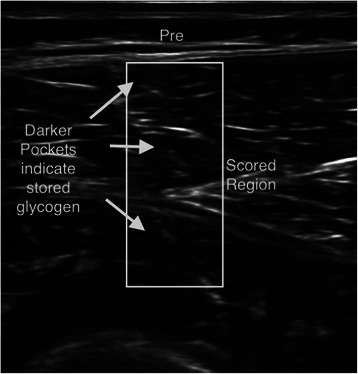
Pre-exercise muscle with high glycogen stores display darker pixel intensities.

**Figure 3:**
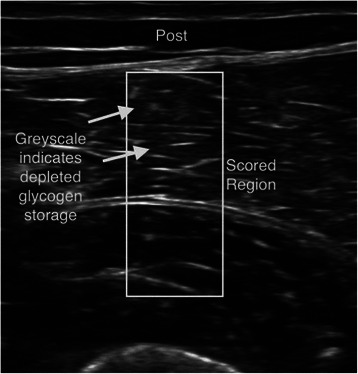
Post-exercise muscle with lower glycogen stores display brighter pixel intensities.

**Figure 4:**
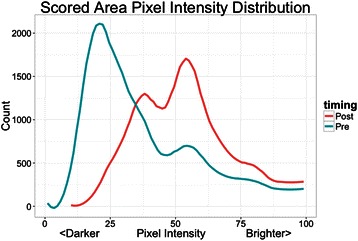
The pixel intensity of the muscle fibers was measured to quantify the amount of glycogen stores within the region of interest. The mean pixel intensity was averaged from the three cropped and segmented scans, and scaled (0 to 100 scale) to create the glycogen score with MuscleSound® software.

Ultrasound measurements and muscle biopsy samples were taken pre-exercise and within 20 to 30 minutes post-exercise. Specific locations on the vastus lateralis and rectus femoris were marked with indelible ink, followed by three ultrasound measurements at each site by a trained technician using a 12 MHz linear transducer and a standard diagnostic high resolution GE LOGIQ-e ultrasound machine (GE Healthcare, Milwaukee, WI). After calculating statistics on the colorbar to determine the general brightness settings of the machine, images were pre-processed and segmented to isolate the muscle area under analysis using a center crop within the muscle section 25 mm from the top muscle sheath (Figure 1). As shown in Figure 2, pre-exercise muscle with high glycogen stores display darker pixel intensities. Figure 3 shows that post-exercise muscle with lower glycogen stores display brighter pixel intensities. The pixel intensity of the muscle fibers was measured to quantify the amount of glycogen stores within the region of interest (Figure 4). The mean pixel intensity was averaged from the three cropped and segmented scans, and scaled (0 to 100 scale) to create the glycogen score with MuscleSound® software.

### Muscle biopsy procedures

Following the ultrasound scans, pre- and post-exercise muscle biopsy samples were acquired on the same leg at the same vastus lateralis locations (2 cm apart). Local anesthesia (1% xylocaine, Hospira, Inc., Lake Forest, IL) was injected subcutaneously. After a small incision, a muscle biopsy sample was obtained using the suction-modified percutaneous needle biopsy procedure [14]. Muscle was trimmed of connective tissue and fat and immediately frozen in liquid nitrogen. Samples were stored at −80°C until subsequent analysis. A glycogen assay kit (Catalog #MAK016, Sigma-Aldrich, St. Louis, MO) was used to determine the concentration of glycogen in vastus lateralis muscle homogenates. In this coupled enzyme assay, glucoamylase hydrolyzed glycogen to glucose, and then the glucose was oxidized to yield a product that reacted with a probe to generate a color detectable with a microplate reader (Synergy H1 Hybrid Reader, BioTek Instruments, Inc., Winooski, VT) at 570 nm.

### Statistical analysis

Data are expressed as mean ± SD. Pre- and post-exercise data were tested for change using paired t-tests, with Pearson correlations used to test relationships between MuscleSound® glycogen scores and muscle glycogen content measured through biochemical techniques.

## Results

Table 1 summarizes subject characteristics, Table 2 performance outcomes, and Table 3 data from the blood samples. The 20 cyclists completed the 75-km cycling time trial in 168 ± 26.0 minutes. Table 2 indicates that oxygen consumption (taken at 16 and 55 km) averaged 69.6 ± 10.3% VO_2max_, with a heart rate of 160 ± 11.5 bpm (89.4 ± 5.9% maximal heart rate). Power output was measured continuously, and averaged 193 ± 57.8 watts, representing 54.2 ± 9.6% watts_max_ on the mountainous course. Subjects reported an RPE of 12.4 ± 1.5 at 15 minutes, 13.1 ± 1.5 at 60 minutes, 14.6 ± 1.8 at 120 minutes, and 17.6 ± 0.7 (“very hard”) at the end of the 75-km cycling trial. Serum cortisol increased 165%, serum myoglobin 654%, and plasma lactate 108% (Table 3), providing further support that the subjects engaged in an intensive and prolonged exercise bout.

**Table 1:** **Subject characteristics (N = 20)**

Variable	Mean±SD
Age	38.4 ± 6.0
Height (m)	1.82 ± 0.7
Weight (kg)	83.3 ± 7.2
Body fat (%)	20.3 ± 5.9
VO_2max_ (ml^.^kg^.-1^ min^−1^)	47.9 ± 7.8
Maximal heart rate (beats/min)	179 ± 8.6
Watts_max_	351 ± 57.6
Maximal ventilation (L/min)	128 ± 17.1
Maximal respiratory rate (breaths/min)	46.7 ± 7.4
Training (km/wk)	154 ± 93.5

**Table 2:** **Performance variables averaged for entire 75 km cycling time trial**

Variable	Mean±SD
VO_2_ (ml^.^kg^.-1^ min^−1^)	33.2 ± 6.4
VO_2_ (%VO_2max_)	69.6 ± 10.3
Watts	193 ± 57.8
% Watts_max_	54.2 ± 9.6
HR (beats/min)	160 ± 11.5
%HR_max_	89.4 ± 5.9
Ventilation (L/min)	74.0 ± 16.7
Rating Perceived Exertion	14.7 ± 1.6

**Table 3:** **Cortisol, myoglobin, lactate, and glucose data from blood samples (mean ± SD)**

Variable	Pre-75 km cycling	Post-75-km cycling	P-value
Serum cortisol (μg/dl)	10.7 ± 4.3	28.4 ± 10.5	<0.001
Serum myoglobin (ng/mL)	32.1 ± 14.7	242 ± 216	<0.001
Plasma lactate (mmol/L)	0.97 ± 0.3	2.02 ± 1.0	<0.001
Plasma glucose (mmol/L)	3.88 ± 0.78	4.25 ± 0.75	0.185

**Figure 5:**
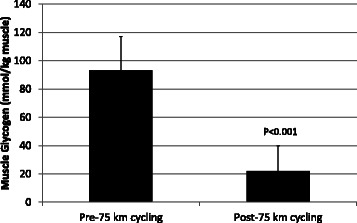
Vastus lateralis muscle glycogen content data pre- and post-exercise, indicating a 77.2 ± 17.4% decrease and an absolute change of 71.4 ± 23.1 mmol glycogen per kilogram of muscle (P < 0.001), as measured by biochemical assay.

Muscle glycogen decreased 77.2 ± 17.4% (Figure 5), with an absolute change of 71.4 ± 23.1 mmol glycogen per kilogram wet weight of muscle (P < 0.001). The absolute change in muscle glycogen varied substantially between subjects (32 to 110 mmol/kg).

**Figure 6:**
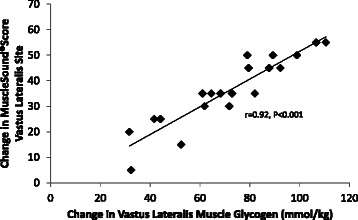
Correlation of the change in vastus lateralis MuscleSound® glycogen score with change in vastus lateralis muscle glycogen content (r = 0.92, P < 0.001).

**Figure 7:**
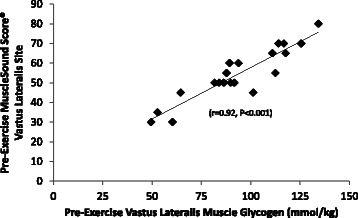
Correlation of pre-exercise vastus lateralis MuscleSound® scores and vastus lateralis muscle glycogen content (r = 0.92, P < 0.001).

**Figure 8:**
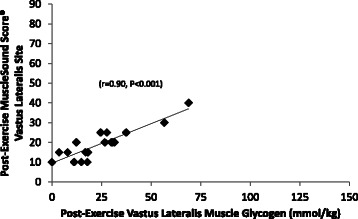
Correlation of post-exercise vastus lateralis MuscleSound® scores and vastus lateralis muscle glycogen content (r = 0.90, P < 0.001).

The MuscleSound® change score at the vastus lateralis site correlated highly with change in vastus lateralis muscle glycogen content (r = 0.92, P < 0.001) (Figure 6). The MuscleSound® change score at the rectus femoris site also correlated highly with change in vastus lateralis muscle glycogen content (r = 0.87, P < 0.001) (data not shown). Figures 7 and 8 indicates that strong, positive correlations were measured for vastus lateralis MuscleSound® scores and muscle glycogen content for pre-exercise and post-exercise time points (r = 0.92, r = 0.90, respectively, P < 0.001).

## Discussion

The 20 cyclists completed the mountainous 75-km cycling time trial in an average of 2.8 hours at a power output of 54% watts_max_ Serum cortisol and plasma lactate increased 165% and 108% in response to this intensive and prolonged exercise bout, and the cyclists experienced an average decrease of approximately three-fourths of glycogen content in the vastus lateralis, as determined directly with pre- and post-exercise skeletal muscle biopsies. The absolute decrease in muscle glycogen content varied widely between subjects. MuscleSound® glycogen change scores acquired non-invasively from an average of three ultrasound scans at the vastus lateralis and rectus femoris sites correlated significantly with change in vastus lateralis muscle glycogen content. Additionally, pre- and post-exercise MuscleSound® glycogen scores were highly correlated with direct muscle glycogen measurements.

These data support the use of the MuscleSound® system for accurately estimating quadriceps muscle glycogen content and exercise-induced decreases in muscle glycogen content despite the wide variation in glycogen depletion following the rigorous 75-km cycling time trial. Hill et al. [13] reported a correlation of 0.81 between change in muscle glycogen content obtained from rectus femoris biopsy samples and the MuscleSound® glycogen change score in 22 cyclists following 90 minutes of steady-state exercise. Glycogen change in the Hill et al. [13] study was modest, and an ultrasound-guided muscle biopsy technique was used to access the rectus femoris without compromising major vascular structures. Muscle biopsy samples are more easily acquired from the vastus lateralis [14], and our data support that ultrasound scans taken at both the vastus lateralis and rectus femoris correlate strongly with change in muscle glycogen content within the vastus lateralis. Few studies have compared exercise-induced glycogen depletion simultaneously in the vastus lateralis and rectus femoris. Kim et al. [15] showed that functional electrical stimulation (FES) but not voluntary dynamic unilateral knee-extensor exercise for 60 minutes decreased muscle glycogen in both the vastus lateralis and rectus femoris to a similar extent. We did not obtain muscle biopsies from the gastrocnemius muscle, a site often used in studies evaluating skeletal muscle glycogen change in runners [16]. Additional research with runners is needed to determine if our muscle glycogen and MuscleSound® data from the vastus lateralis in cyclists can be extrapolated to the gastrocnemius muscle in long distance runners.

Muscle glycogen is the primary source of fuel during prolonged and intensive exercise, and the relationship between muscle glycogen and fatigue resistance is supported through several lines of experimental evidence including alterations in pre-exercise muscle glycogen content by dietary and exercise interventions [1-3,16,17], and the development of profound fatigue during exercise in individuals with McArdle disease which restricts glycogen metabolism [18]. As demonstrated in the current study, glycogen depletion rates during exercise vary widely between athletes, even when duration and intensity are controlled, and this could be due to multiple factors including variance in pre-exercise muscle glycogen levels and ability to beta-oxidize fatty acids [2,3,13]. Use of the suction-modified Bergström percutaneous needle biopsy technique to obtain skeletal muscle tissue samples from the vastus lateralis of human subjects imposes significant subject burden, typically requires physician involvement and oversight, and is costly in terms of supplies and personnel [14]. These barriers are largely removed through utilization of high frequency musculoskeletal ultrasound for non-invasive muscle glycogen assessment with the MuscleSound® system.

MuscleSound® methodology is based upon measurement of the water content associated with glycogen in the muscle. This study and that of Hill et al. [13] support a strong correlation between ultrasound- and biochemical-based measurements of skeletal muscle glycogen. Additional research is needed to determine how exercise-induced changes in muscle water content influence this relationship. Skeletal muscle water content can vary depending on the hydration status of the athlete, and the influence of acute exercise and disease states [10,11]. Ultrasound velocity in the soleus muscle has been shown to correspond to changes in urine osmolarity and specific gravity during acute dehydration and rehydration in collegiate wrestlers [19]. The strong positive correlations demonstrated in our study between MuscleSound® glycogen scores and vastus lateralis glycogen content suggests that exercise-induced alterations in muscle tissue hydration has little effect on the pixel intensity used to calculate the glycogen score.

## Conclusions

In this validation study, MuscleSound® change scores acquired from an average of three ultrasound scans at the vastus lateralis site correlated significantly with change in vastus lateralis muscle glycogen content assessed through a biochemical assay. These data extend the findings of Hill et al. [13] and support the use of the MuscleSound® system to accurately and non-invasively estimate exercise-induced decreases in vastus lateralis skeletal muscle glycogen content. Further research is needed with additional muscle groups and a wide variety of athletes under varying environmental conditions to confirm the within-subject and between-subject value of using ultrasound scans for muscle glycogen determination.

## Abbreviations

ECLIA: Electrochemiluminescence immunoassay
EDTA: Ethylenediaminetetraacetic acid
glycoCEST: Use of the water signal by magnetic resonance spectroscopy for glycogen analysis
MRS: Magnetic resonance spectroscopy
RPE: Rating of perceived exertion
